# Draft Whole-Genome Sequence of a Catalase-Negative *Staphylococcus aureus* subsp. *aureus* (Sequence Type 25) Strain Isolated from a Patient with Endocarditis and Septic Arthritis

**DOI:** 10.1128/genomeA.01442-16

**Published:** 2016-12-22

**Authors:** Byron Berenger, Justin Chen, Anne-Marie Bernier, Kathryn Bernard

**Affiliations:** a Alberta Provincial Laboratory for Public Health, Edmonton, Alberta, Canada; b Division of Infectious Diseases, University of Alberta, Edmonton, Alberta, Canada; c Department of Biology, Université de Saint-Boniface, Winnipeg, Manitoba, Canada; d National Microbiology Laboratory, CSCHAH Site, Public Health Agency of Canada, Winnipeg, Manitoba, Canada; e Department of Medical Microbiology, University of Manitoba, Winnipeg, Manitoba, Canada

## Abstract

*Staphylococcus aureus* strains without catalase activity are rare, challenging to identify with conventional biochemical methods, and, despite a supposed decreased pathogenicity, can still cause disease. The first whole-genome sequence of a catalase-negative *S. aureus* isolate causing severe recurrent invasive infection with two novel missense mutations in the *katA* gene is reported here.

## GENOME ANNOUNCEMENT

Catalase protects bacteria from hydrogen peroxide and is used in the phenotypic identification of *Staphylococcus aureus* (1, 2). Despite a potential role in pathogenicity, catalase-negative isolates can cause infections (3–5). Sequencing of the *katA* gene has revealed multiple mutations/deletions responsible for this phenotype (6–8), but no whole-genome sequence has been performed on catalase-negative isolates of this important pathogen.

A catalase-negative (3% H_2_O_2_), methicillin-susceptible *S. aureus* subsp. *aureus* (CN-MSSA) was isolated from a patient with bacteremia, native aortic valve endocarditis, and bilateral knee native joint septic arthritis. The patient’s past medical history included diabetes and hypertension. CN-MSSA was isolated from blood, the aortic valve, and knee synovial fluid; the bacterium grew well in air at 35°C in 24 h, with golden-pigmented, beta-hemolytic colonies which were tube- and slide-coagulase positive (9). Identification (Vitek MS and Vitek 2; bioMérieux) and cefazolin disk susceptibility testing on Mueller-Hinton agar (Oxoid) were done in a CAP-accredited laboratory following CLSI guidelines. The isolate was susceptible to all other antibiotics tested, except for penicillin (data not shown). The patient received knee washouts on post-admission day (PAD) 3 (growth) and PAD 17 (no growth) and a bioprosthetic aortic valve replacement on PAD 9. Blood cultures remained positive until PAD 12 despite high-dose cloxacillin therapy. The patient received a six-week course of cloxacillin (2 g IV q4h) and rifampin post-surgery. Five days after completing the antibiotic course, the patient presented with relapsed CN-MSSA bacteremia, development of an aortic root abscess, and new mitral and tricuspid valve vegetations. This required a homograft replacement of the aortic root and valve and repair of the tricuspid and mitral valves. There was no evidence of metastic infection at that time. The CN-MSSA grew again from blood and the excised bioprosthetic valve tissue. After the second surgery the patient was treated with cefazolin and rifampin for 10 weeks. An isolate from the aortic valve removed during the first surgery was used for whole-genome shotgun sequencing.

A whole-genome shotgun paired-end library was prepared, sequenced, and assembled as previously described (10). This generated 1,221,050 reads with a total of 366,411,852 bp on a MiSeq sequencer (Illumina) and produced a draft genome of 2,782,699 bp in eight contigs (>1,000 bp) with 142-fold coverage and a 32.68% G+C content. Annotation of the genome using Prokka (11) revealed 2,599 coding sequences, 58 tRNA genes, and nine rRNA sequences. The isolate was sequence type 25, based on *in silico* multilocus sequence type analysis (pubmlst.org).

The catalase gene and protein sequence from National Microbiology Laboratory (NML) identifier 151290 were compared to *S. aureus* NCTC 8532^T^ using BLAST. Two silent mutations were identified at C1146T and C1239T, and two novel missense point mutations were identified at T490C (Y164R) and T549A (D183E). The Y164R mutation may interfere in the tetramer binding capacity as it is situated between two amino acid residues (162 and 165) that are essential for this functional domain of the protein (12). The second point mutation (D183E) is in the NADPH-binding domain and could also contribute to the inactivation of the enzyme.

### Accession number(s).

The draft genome of strain NML 151290 has been deposited at DDBJ/EMBL/GenBank under the accession number MEGZ00000000. The *S. aureus* NCTC 8532^T^ accession number used was WP_000082539.1.

## References

[1] Das D, Bishayi B 2009 Staphylococcal catalase protects intracellularly survived bacteria by destroying H2O2 produced by the murine peritoneal macrophages. Microb Pathog 47:57–67. doi:10.1016/j.micpath.2009.04.012. 19439176

[2] Das D, Saha SS, Bishayi B 2008 Intracellular survival of *Staphylococcus aureus*: correlating production of catalase and superoxide dismutase with levels of inflammatory cytokines. Inflamm Res 57:340–349. doi:10.1007/s00011-007-7206-z. 18607538

[3] Ellis MW, Johnson RC, Crawford K, Lanier JB, Merrell DS 2014 Molecular characterization of a catalase-negative methicillin-susceptible *Staphylococcus aureus* subsp. *aureus* strain collected from a patient with cutaneous abscess. J Clin Microbiol 52:344–346. doi:10.1128/JCM.02455-13. 24131694 PMC3911441

[4] Horiuchi K, Matsumoto T, Hidaka E, Kasuga E, Sugano M, Oana K, Kawakami Y, Honda T 2012 Isolation and molecular characterization of catalase-negative *Staphylococcus aureus* from sputum of a patient with aspiration pneumonia. Jpn J Infect Dis 65:439–441. doi:10.7883/yoken.65.439. 22996221

[5] Kallstrom G, Chang T, Albertson M, Morilla D, Fisher MA, Eberly B 2011 Recovery of a catalase-negative *Staphylococcus epidermidis* strain in blood and urine cultures from a patient with pyelonephritis. J Clin Microbiol 49:4018–4019. doi:10.1128/JCM.01031-11. 21900516 PMC3209119

[6] Lagos J, Alarcón P, Benadof D, Ulloa S, Fasce R, Tognarelli J, Aguayo C, Araya P, Parra B, Olivares B, Hormazábal JC, Fernández J 2016 Novel nonsense mutation in the *katA* gene of a catalase-negative *Staphylococcus aureus* strain. Braz J Microbiol 47:177–180. doi:10.1016/j.bjm.2015.11.012. 26887242 PMC4822749

[7] Teo JW, Kum S, Jureen R, Lin RT 2015 Molecular characterization of a catalase-negative *Staphylococcus aureus* blood culture isolate. J Clin Microbiol 53:3699–3701. doi:10.1128/JCM.01271-15. 26354811 PMC4609694

[8] To KK, Cheng VC, Chan JF, Wong AC, Chau S, Tsang FH, Curreem SO, Lau SK, Yuen KY, Woo PC 2011 Molecular characterization of a catalase-negative *Staphylococcus aureus* subsp. *aureus* strain collected from a patient with mitral valve endocarditis and pericarditis revealed a novel nonsense mutation in the *katA* gene. J Clin Microbiol 49:3398–3402. doi:10.1128/JCM.00849-11. 21715584 PMC3165592

[9] Becker K, Skov RL, Von Eiff C 2015 *Staphylococcus*, *Micrococcus*, and other catalase-positive cocci., p 354–382. *In* Jorgensen JH, Pfaller MA, Carroll KC, Funke G, Landry ML, Richter SS, Warnock DW, Warnock DW (eds), Manual of clinical microbiology, 11th ed, vol 1 ASM Press, Washington, DC.

[10] Bernier AM, Bernard K 2016 Draft genome sequence of *Trueperella bernardiae* LCDC 89-0504^T^, isolated from a human blood culture. Genome Announc 4(1):e01634-15. doi:10.1128/genomeA.01634-15. 26950329 PMC4767919

[11] Seemann T 2014 Prokka: rapid prokaryotic genome annotation. Bioinformatics 30:2068–2069. doi:10.1093/bioinformatics/btu153. 24642063

[12] Marchler-Bauer A, Derbyshire MK, Gonzales NR, Lu S, Chitsaz F, Geer LY, Geer RC, He J, Gwadz M, Hurwitz DI, Lanczycki CJ, Lu F, Marchler GH, Song JS, Thanki N, Wang Z, Yamashita RA, Zhang D, Zheng C, Bryant SH 2015 CDD: NCBI's conserved domain database. Nucleic Acids Res 43:D222–D226. doi:10.1093/nar/gku1221. 25414356 PMC4383992

